# Sector sensor array technique for high conductivity materials imaging in magnetic induction tomography

**DOI:** 10.1186/s12938-019-0734-2

**Published:** 2019-12-02

**Authors:** Jia Chen, Li Ke, Qiang Du, Wanni Zu, Xiaodi Ding

**Affiliations:** Institute of Biomedical and Electromagnetic Engineering, Shenyang University of Technology, Shenyang, China

**Keywords:** Magnetic induction tomography, Sector array, Forward electromagnetic problem, Sector magnetic induction tomography (SMIT)

## Abstract

**Background:**

Magnetic induction tomography (MIT) is a tomographic imaging technique, which has potential applications in security, industry, and medicine. Typically, sensors form a closed structure around the object. However, the measurement cannot be achieved using a closed sensor array in the process of severe brain trauma nursing and the neurosurgery operation.

**Results:**

The new sector sensor array magnetic induction tomography (SMIT) system is developed to realize real-time monitoring in the treatment of the brain. The functions of the drive coil and the sensor coil are separated in this system. The detection sensitivity of the imaging region boundary is analyzed through simulation. The sensor array locates on the high detection-sensitivity area, and the low sensitivity detection area is reserved for operation and clinical equipment. The sensor array received the energy of the signal accounts for reach 90% of the total energy. The integrity measuring data are obtained using a rotating scan in the system. In the experiment, we analyze the effects that system parameters have on the quality of imaging, for example, the scan step size, the number of sensors, the coverage angle of the sensor array and the scan angle. The experiment result provides a reference for the SMIT system design under a particular condition. In the complete measurement, the SMIT system reconstructs the images of center goal and margin goal, and the actual images have high peak signal-to-noise ratio.

**Conclusions:**

The SMIT system can rebuild the conductivity distribution of the imaging region using incomplete space. In rotation measurement, the system provides a working place for clinical care. The flexible design of the system based on the experiment result makes the different treatment for brain injury own matched SMIT equipment.

## Background

Magnetic induction tomography (MIT) is a tomographic imaging technique based on the mutual inductance of coils. This imaging technique is non-touch and noninvasive, and the sensor coils locate around the target [1, 2]. The ROI (region of interest) exposes to alternating magnetic fields, and the eddy currents are induced in the object. These eddy currents generate a secondary magnetic field (∆**B**), and this magnetic field contains information about the conductivity distribution inside the object. The phase change between the primary magnetic field (**B**) and the total magnetic field (**B **+ ∆**B**) [3–5] can be obtained from the phase difference between the drive coil and the sensor coils. The goal images can be constructed based on the measurement data of the sensor at each position.

In the past few years, MIT has been primarily developed for medical imaging applications such as imaging brain function or stroke detection [6]. Previous MIT mainly used fixed coils array, and all coils are located around the periphery of the imaging area. Each coil of the system is both of a sensor coil and a drive coil [7–10]. The MIT systems achieve circumference measurement by switching the functions of coils. The continuous monitoring function of MIT has an advantage in the treatment of traumatic brain injury, however, the brain surgical operations and the wound care unable to supply suitable space for the typical MIT system. It means that the sensors need to give way to the treatment and care in the place of the wound (i.e. the coils cannot take operating area). The system cannot achieve the monitoring of the brain without a complete sensor array in these cases. In the limited-angle MIT imaging study, a part of the coil array (these coils have exciting and detection functions) has been directly abandoned. The incomplete measurement images cannot be used as a diagnostic basis. So, the sensor array design needs to achieve complete measurement, and the system can provide enough operating region simultaneously. The distribution of the drive magnetic field is a sector in the imaging area. This feature makes the sensor array can locate high-sensitivity region, and the other area set as the operating region. Therefore, the design strategy of sensor distribution is proposed based on the simulation analysis results of edge magnetic induction. The sensor array locates on the space where the magnetic field has high information effectiveness.

**Fig. 1:**
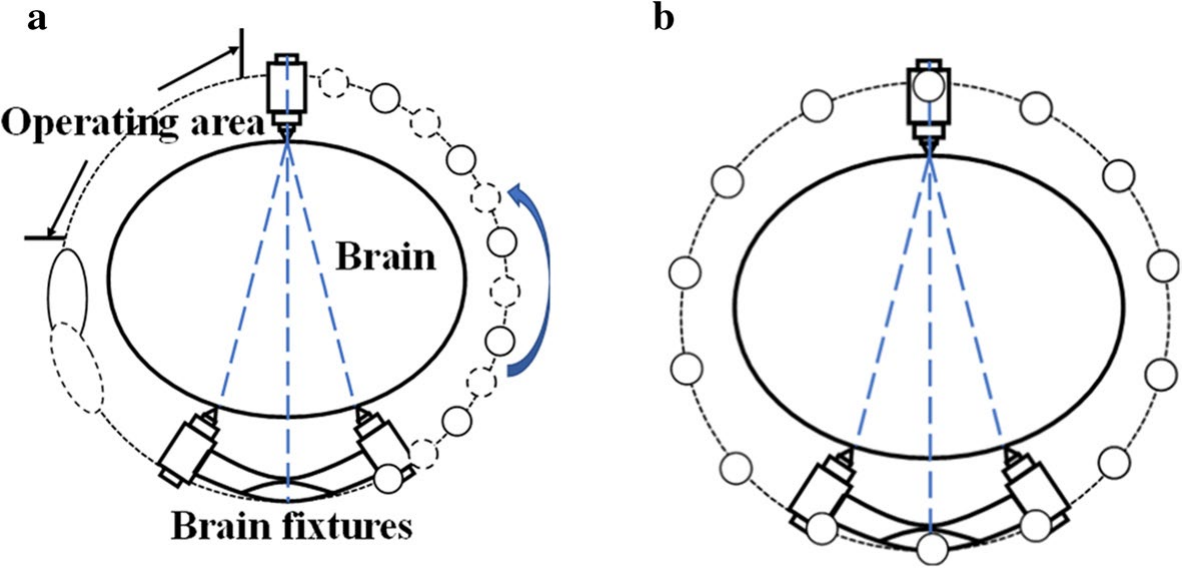
The locating place of the sector sensor array (**a**) and the typical sensor array (**b**) in brain surgery. In the SMIT sensor array, the space between the drive coil and the sensor can be used as the operating area for doctors. The operating area is occupied only during the process of the scan. The repetitive installation of the sensor array can be avoided during surgery, in contrast to the classical MIT system. This feature can ensure the positioning accuracy of tomography

In this paper, a sector sensor array MIT system (SMIT) scheme is proposed to solve non-holonomic sensor array detection. In this system, the sensor array is designed as half-round based on the results of magnetic field calculation. The drive function of the coil is separated from the sensor array, and the big drive coil can generate a strong primary magnetic field [11]. The sensor coil array and drive coil are located on opposite sides of the imaging field. The structure of the sensor array makes the measured magnetic field has more information about the goal. In the complete measurement, the rotation is accomplished by a rotating platform in the imaging device. The comparison diagram of MIT and SMIT in craniocerebral operation is shown in Fig. 1. The sector sensor array can provide manipulation space for doctor compared to MIT. We do the single goal imaging experiments at different points, and the system parameters experiment is done to test the imaging quality of the system with different setups. Experimental results show many factors affect imaging quality, including the number of sensors, scan angles and rotating scan using different step angles. The imaging experiments under different conditions have been done to verify the performance and reliability of the system. In the complete circumference scan experiment, the SMIT system can detect the location of the goal.

## Results

**Fig. 2:**
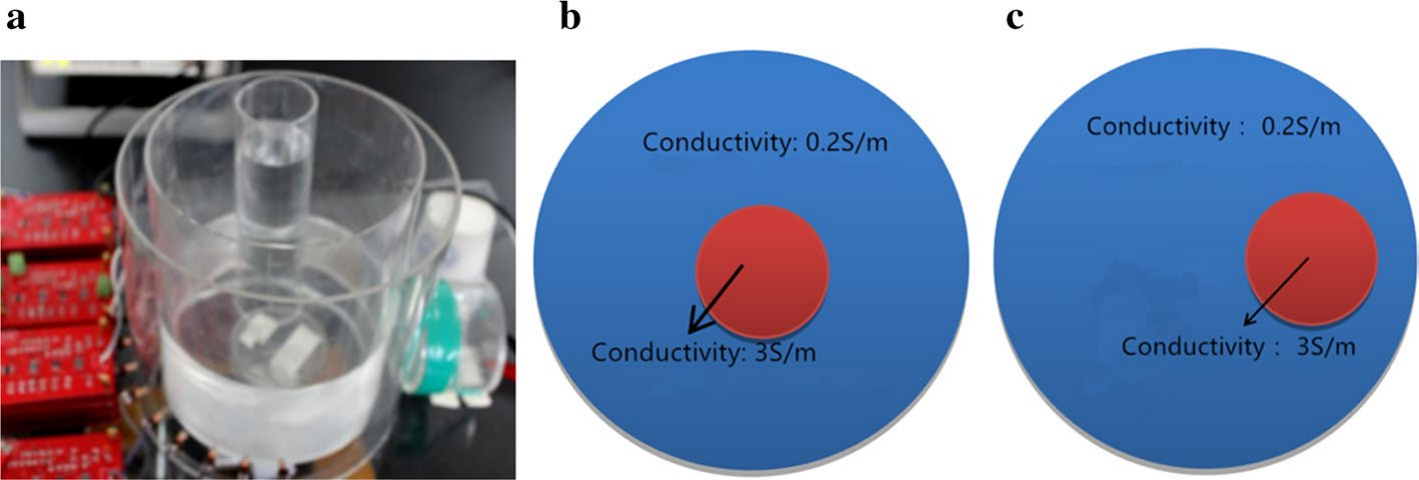
The imaging field and the location of the goal. **a** The imaging region with a goal, the NaCl solution in the bigger beaker is the background of images, and the NaCl solution in small beaker is the goal. **b** The diagram of center goal. **c** The diagram of the distal goal

The simulation study shows that the SMIT scheme using the specific structure sensor array has better sensitivity in measuring the phase change. The imaging experiments in two conditions are completed to prove the feasibility of the system further. The image reconstruction test includes the center goal (CG) and marginal goal (MG) experiments. In the actual system, a cylindrical insulating container is used as the imaging region (see Fig. 2a). It has an external diameter of 160 mm, and the inside diameter is 154 mm. The saline solution (0.2 S/m) is injected into the container as the imaging background, and the height of the solution is 100 mm. The imaging goal is an insulating transparent cylindrical container with an external diameter of 50 mm, and the inside diameter is 46 mm. The conductivity of the solution in the goal container is 3.0 S/m.

The parameters of the system can affect the quality of imaging in the process of measuring. In the first experiment, four experimental tests are carried out for verifying the relation between system parameters and images quality. The measurement parameters of the system include the coverage angle of sector, the number of testing channel, the angle of measurement step and the incomplete circumference scanning.

*Test 1* The relation between the covering angle of sector array and the imaging quality. The experimental setup can be seen in Fig. 2. The angle range of sector array: 0° (channel 8), 51° (channels 6–10), 128° (channels 3–13) and 180° (channels 1–15), and the center of sector array located on the 8th channel opposite the drive coil. The size of the scan step is 10°.

Table 1 shows the imaging results of the center goal and marginal goal using different array covering angles. The imaging results are marked as 0°, 51°, 128° and 180°. The change of the sector angle influences the measured accuracy of the center goal boundary, and the broader sector angle, the higher the boundary accuracy. In the edge goal test, the artifacts become more significant when the detecting angle becomes smaller; especially, the goal cannot be detected using 0° sensor array. It means that smaller sensor array cannot prove enough data for imaging.

**Table 1:**
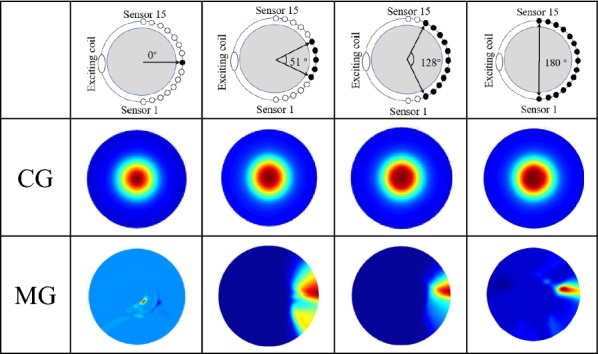
The SMIT system reconstructs Images using different sector sensor array

The black dots represent working electrodes

*Test 2* The number of measuring channel effect on the imaging quality. In the test, the number of channels is: 3 (channel 1, 8, 15), 5 (channel 1, 5, 8, 11, 15), 8 (channel 1, 3, 5, 7, 9, 11, 13, 15) and 15 (all coils of the sensor array). The sensors in the test are distributed evenly in the sector array. The size of the scan step is 10°. The number of measuring data is increased to 15 using linear interpolation to meet the requirement of the imaging algorithm.

Table 2 shows the images of the center goal and marginal goal using different numbers of channels. The imaging results show the imaging ability when the system uses 3, 5, 8, 15 channels. The increase in the number of sensors enhances the quality of images.

**Table 2:**
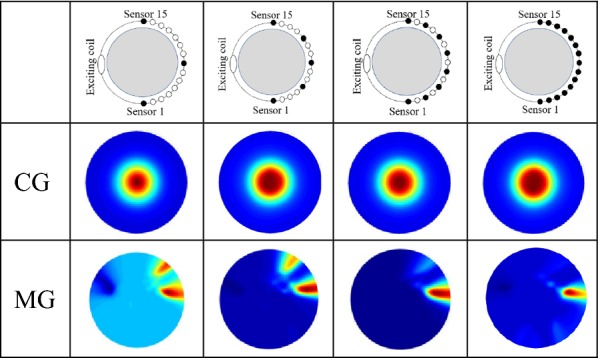
The SMIT system reconstructs images using different electrode density

The electrodes are uniform distribution in the array

*Test 3* The impact of step size on the quality of images. The angle of one step in the test is 20°, 30°, 40° and 60°. The number of measurement data is increased to 15 using linear interpolation.

Table 3 shows the imaging results of the central goal and marginal goal using different angles of one step. Reconstructed images are marked as 20°, 30°, 40°, and 60°. The angle change of the step size has almost no influence on the boundary detection of central goal images. However, the image contrast becomes worse when the step size increase. In the edge goal imaging experiment, the artifacts enlarge when the step size increase. The image has no valid reveal about the goal when the angle of step is 60°.

**Table 3:**
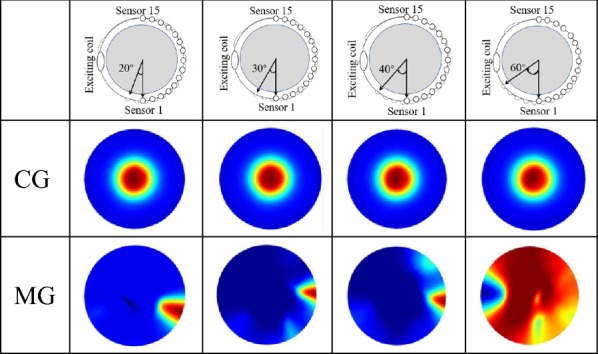
The SMIT system reconstructs images using different scan step size

*Test 4* The effects of finite-angle scan on the quality of imaging. The angle range of scan is 0° (no rotating), 90°, 180°, and 270°, and the size of the step is 10°. The scan process of all tests has a central point (8th channel).

Table 4 shows the imaging results of the goal when the system uses four different scan angles. In no rotating condition, the images can be interpreted as the imaging process with no scan. The images express the goal locates on the path of the magnetic line. The boundary of images become precise with the increase of scan angle. The system can achieve the location of the goal when the scan angle reaches 180°. However, because of the information loss in non-holonomic scan measurement, the boundary of images is blurring.

**Table 4:**
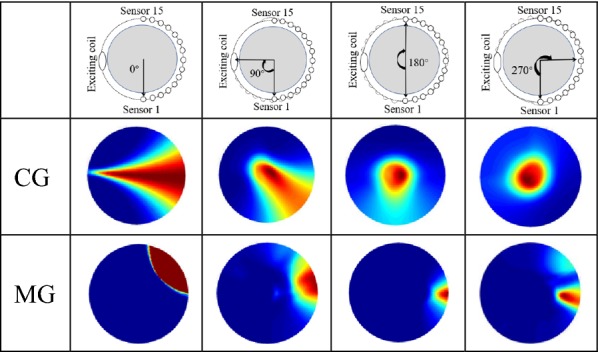
The SMIT system reconstructs images using different scan angle

The scan angle is the rotation angle of the system in one experiment, and all electrodes are used to detect data

In the second experiment, the total scan test is done to verify the imaging feasibility of the system. In the experiment process, the data of the uniform field are measured as the calibration parameters. The transparent cylindrical containers are placed into the imaging field of the SMIT system. The schematic diagrams of the centre goal and marginal goal in the SMIT system is shown in Fig. 2b, c. Thirty-six sets of data are acquired when the rotation angle of one step is 10°, and the data volume is 15 × 36. The imaging results of the centre goal and marginal goal is shown in Table 5. The comparison results of ideal, mimic and actual images using PSNR is shown in Table 6. The PSNR of any two images is calculated to verify the quality of images further. The experiment images and the simulation images are substantial agreement, and the measurement data of the system can satisfy the requirement of images reconstruction.

**Table 5:**
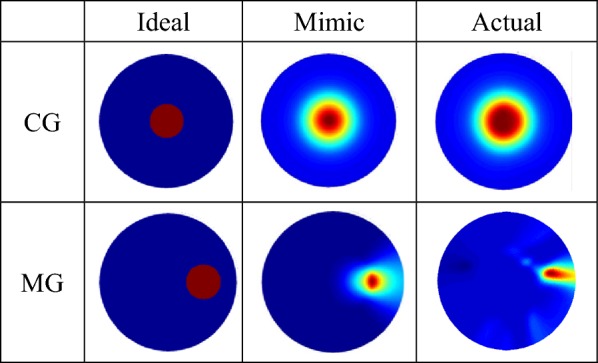
The SMIT system reconstructs images of centre goal and margin goal

The ideal images are the real position of goal in the imaging region

**Table 6:** The PSNR of reconstruction images

	Center goal			Marginal goal		
	Ideal	Mimic	Actual	Ideal	Mimic	Actual
Ideal	–	36.5678	39.9100	–	37.4047	43.8900
Mimic	36.5678	–	31.2587	37.4047	–	40.6959
Actual	39.9100	31.2587	–	43.8900	40.6959	–

In this table, the PSNR is calculated using any two of three images (ideal images, mimic images, actual images) in one experiment

## Discussion

The SMIT system is built to solve the structure issue of MIT system in the clinical application of brain injury monitoring. The system can prove ample space for the operation of treatment and nursing, and the operation of treatment has no impact on continuous monitoring. The design strategy of the coil array follows the actual demand. Some sensor coils are abandoned to achieving the design goal from a technical viewpoint. The conductivity distribution of the detection region can be reconstructed using the incomplete sensor array. The function separation of coil sensors makes the drive coil independent from the sensor coils. In this system, the relative location between the sensor coils and the drive coil is constant. The rotating platform provides complete measurement data for the imaging. The complete measurement experiment verifies the imaging ability and the stability of the SMIT system. In the system parameters experiment, four system parameters have been tested, respectively. In no artificial therapeutic process, the system regularly scans the brain to monitor the advancing of disease. The number of sensor coil and the angle of the scan can be adjusted to meet the requirement of the wound care and brain surgery. The SMIT system can guarantee the consistency of the imaging section. This feature can avoid the effect of the reduplicative install of sensor array on the measurement accuracy. In the more rigorous conditions (for example, the neurosurgery operation), the size of the sensor array and the angle of step also need consideration. The system parameter experiment, in this paper, provides some reference for the further study of SMIT system.

The rotating scan is the characteristic of SMIT system. The fixing of equipment and the stable operation of coil array are the problems to be solved. The rotation mechanism needs to avoid secondary damage to the patient. The system movement will accompany the more complex problem of electromagnetic compatibility. These issues need to be solved before the SMIT system applies to the clinical.

## Conclusions

This paper presents the SMIT system using a sector sensor array to reconstruct the conductivity distribution of the object. The system can complete the measurement process without the closed sensor array. The structure of SMIT provides the MIT with design philosophy when the space of the clinical application is limited. In the system parameter experiment, the imaging results verify several vital parameters of the imaging quality. These experimental results provide reference data for future system design. In the second experiment, the images of complete scan measurement are evaluated by the PSNR. The quality of the actual images is almost consistent with the mimic images. It is anticipated that the sector magnetic induction tomography system can be used for the cerebral injury monitor.

## Methods

### Forward problem

The SMIT model is generated based on the previous forward and inverse MIT arithmetic [5]. The forward problem of MIT is a typical eddy current problem. This problem is solved using an edge finite element method (FEM) and with the aid of magnetic vector potential (**A**). The (**A**, **A**) formulation can be obtained from Maxwell’s equations [9]:

1 $$\frac{1}{\mu }\nabla^{2} {\mathbf{A}} - j\omega \sigma {\mathbf{A}} = {\mathbf{J}}_{{\mathbf{s}}}$$

where *σ* is electrical conductivity, *μ* is magnetic permeability, *ω* is angular frequency, and **J**_**s**_ is the current density of drive coil. Equation (1) can be solved using finite-element methods (FEM), and the magnetic vector potential **A** can be calculated. The curl of **A** is the magnetic induction.

### Back projection algorithm

Based on electromagnetic theory, the conductivity of the imaging region affects the imaginary part of the total magnetic field [12]. The conductivity of the imaging region can be reconstructed using the phase-difference.

The magnetic field lines back-projection algorithm is considered for the image reconstruction [13, 14]. This method is based on the distribution matrix D of the magnetic line. The conductivity distribution of the imaging field multiply by the matrix D, and the result is the phase-difference of boundary magnetic field. In actual measurements, the phase change of the magnetic field is obtained from the phase-difference between the drive signal and the detection signal. The equation is defined as:

2 $$C_{m} = D \cdot F_{n}$$

where *C*_*m*_ is the change vector of conductivity, *m* is the sum of pixels in the back-projection image region, *F*_*n*_ is the standardized data of boundary phase-difference, and *D* is an (*m* × *n*) magnetic line distribute matrix.

In the process of image reconstruction, the rotation angle and boundary phase-difference need to be measured. The distribution matrix of the magnetic lines multiply by the standardized phase-difference data and the result is a series of phase change matrix. The phase change matrix rotates the same angle in the opposite direction and superposed together, and the reconstruction image can be obtained. In the process of measurement, *n* is the number of steps of rotation, *θ* is the rotation angle of every step. The equation of the back-projection algorithm is defined as:

3 $$C\left( m \right) = \sum\limits_{i = 1}^{n} {{\text{rotate}}\left( {\mathop \sum \limits_{j = 1}^{N} D\left( {m,j} \right)\frac{{\varphi_{u} \left( {i,j} \right) - \varphi_{f} \left( {i,j} \right)}}{{\varphi_{f} \left( {i,j} \right)}},n \times \theta } \right)}$$

where *C*(*m*) is the reconstructed image matrix, *X* is the sum of rotating steps, *j* is the number of boundary sensor coils, the rotate function is used to spin a matrix in the counterclockwise direction, the *n* × *θ* is the rotation angle.

### Image quality assessment

The peak signal-to-noise ratio (PSNR) is used as an image quality assessment method in the image reconstruction system [15]. The PSNR is the mathematical measure of image quality based on the pixel, and it expresses the effect of discrepancies between two images on the total quality of images. The PSNR has a high value when two images have preferable consistency in the corresponding pixel. In the next chapters, PSNR will be used to evaluate the quality of the reconstructed image. The PSNR is calculated using the equations in [16]; it is defined as follows:

4 $${\text{PSNR}} = 10\log_{10} \frac{1}{D}$$

In Eq. (4) the $D$ is defined as

5 $$D = \frac{1}{M \times N}\mathop \sum \limits_{i = 0}^{M - 1} \mathop \sum \limits_{j = 0}^{N - 1} \left( {x\left( {i,j} \right) - \hat{x}\left( {i,j} \right)} \right)^{2}$$

where *M* and *N* is the width and height of the image, *x* (*i*, *j*) is the normalization value of the standard image in point (*i*, *j*), *x* is the grayscale values of reconstruction image. The unit of PSNR is dB. The PSNR is zero when two images are entirely different, and the PSNR of the two same images is ∞. It means that images have a high PSNR when the system restore conductivity distribution accurately. In this paper, the ideal image is designed as the standard image. The background value of the standard image is set to 0, and the pixel value in the goal region is 1. The PSNR is calculated between any two of the three images (standard images, simulated images and the real images). The result of PSNR calculation is used as the primary basis for the system performance assessment.

### Simulation analysis

The system simulation modelling approach is realized in two steps: the definition of a physical problem and the establishment of the solid model. In 3D MIT system model, the brain is the eddy field and imaging region. The brain is also covered by the fields generated by the drive coil. The air field is not an eddy field, and the scope of air field is big enough to guarantee the attenuation of the magnetic field. The MIT simulation is a 3D eddy current field analysis, to be convenient, we make the following assumptions: The current density is the forms of the sine function; ignore the displacement current; ignore the effect of temperature on the conductivity; the conductivity of brain tissue is constant.

**Fig. 3:**
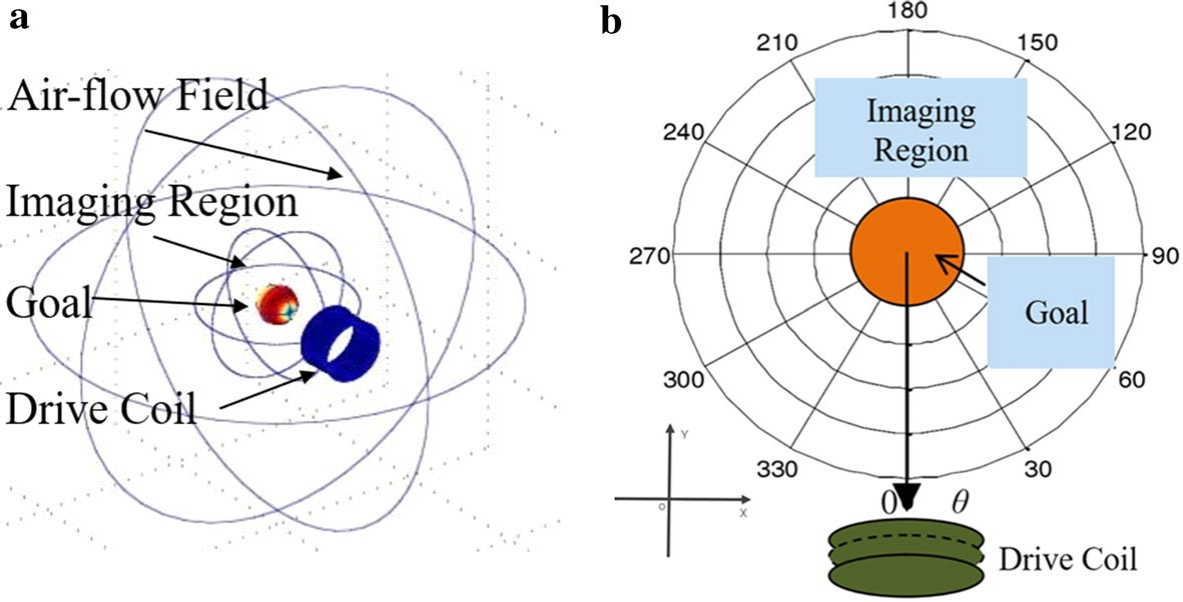
The simulation model of SMIT in FEM software and the data acquisition plane in the model. **a** The simulation geometry model for SMIT in software. **b** The images data acquisition region of SMIT system. The sign points at the edge of the imaging region are used to confirm the location of coils

The simulation model of SMIT is built using the commercial FEM package (COMSOL) (Fig. 3a). In the brain model, the brain and the focus are set as the sphere. The geometrical feature of the model is described in the software. In this example, the SMIT model includes drive coil, perturbation, brain, and air field. The drive coil with a radius of 100 mm locates on the boundary of the imaging region. The solution domain is a ball with a radius of 20 cm, and the boundary is magnetic insulation. The frequency of the drive signal is 8 MHz, and the current density is 25 A/cm^2^. The parameter of mesh generation is 0.005, and the unit growth rate is 1.5. The AC/DC module is used to solve the quasi-static magnetic field. The solver is the GMRES linear solver.

According to the fundamental of MIT, the exciting coil provides the main magnetic field (**B**) in the space. The Internal eddy of the object generates the secondary field (∆**B**). The perturbation distribution can be reconstructed based on the phase change between the main field B and the total field (B + ∆**B**). The phase change can be acquired by measuring the phase-difference of the potential between the driver coil and the sensor coils [4]. In this paper, the measurement sensitivity of the sensor region is analyzed by phase-difference value.

**Fig. 4:**
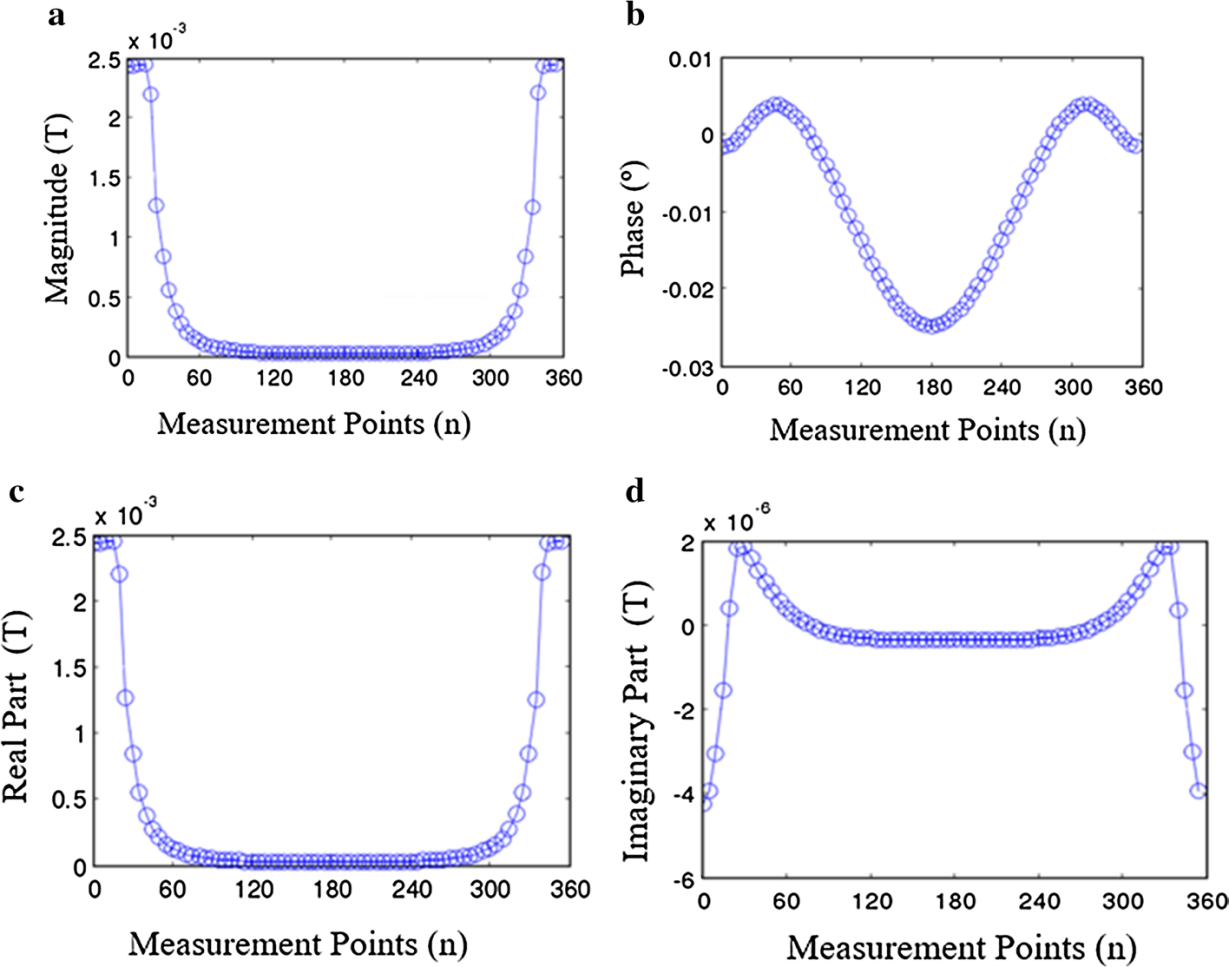
The magnetic induction intensity analysis of different points in the imaging field boundary. **a** The magnitude of the magnetic induction intensity. **b** The phase-difference between the drive signal and the measured signal. **c** The real part of the magnetic induction intensity. **d** The imaginary part of the magnetic induction intensity

In the MIT, the imaging region is a 2D section among the 3D object. The simulated analysis of system uses the 2D SMIT model (0.2 S/m) to calculate the boundary phase-difference (as in Fig. 3b). In the 2D model, the imaging region locates on the *X*–*Y* plane of the 3D model. The sensor coils are placed on the boundary of the imaging domain. The serial number on the edge of the imaging domain is used to mark the location of the measuring points. The model has a radius of 100 mm, and the perturbation (has a radius of 10 mm) at the point (0, 0). The change of magnetic induction in the measuring points directly express the information about perturbation. The phase-difference data are obtained from the simulation model. In the process of simulation, the conductivity of perturbation changes from 0.2 to 2 S/m, the increment of every step is 0.2 S/m. The imaging domain is a uniform field when the conductivity of the perturbation is 0.2 S/m. By analyzing the uniform field, the distribution of magnetic induction is expressed by the simulation data of detection points. The simulation results of magnetic induction parameters in the uniform field are shown in Fig. 4. The variable on the horizontal axis is the θ (see in Fig. 3b) which show the location of measurement points. The magnitude of the magnetic induction is shown in Fig. 4a; meanwhile, the phase-difference, real part and imaginary part are shown in Fig. 4b–d. The magnitude of the magnetic induction much reflects the energy distribution of the magnetic field. The maximum of amplitude locates near 0° point (i.e. images reconstruction using magnetic induction is infeasible). The curve of the real part is similar to the magnetic induction, and the imaginary part has a negative maximum at the place of the exciting coil. The uniform field impacts the distribution of phase-difference of the region of 90°–270°. It means that the phase signal with imaging field information is retentive with the attenuation of the magnetic field. Therefore, the phase-difference can read conductivity distribution of the magnetic field.

**Fig. 5:**
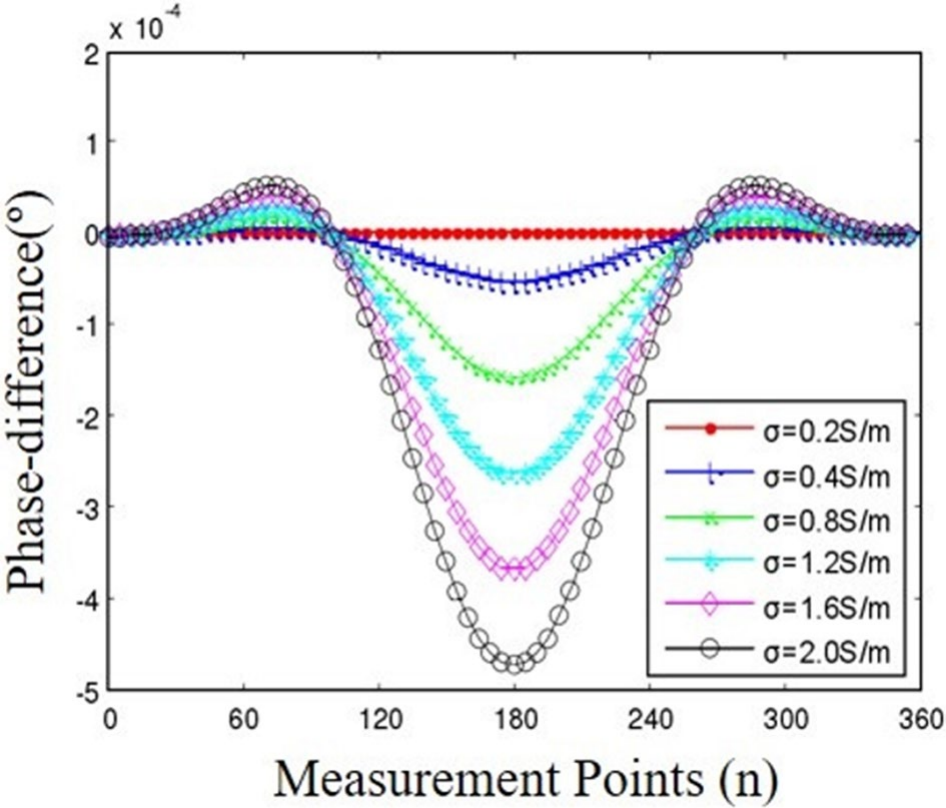
The boundary phase-difference distribution of the imaging region. The simulation results show the phase-different has high sensitivity to the conductivity change of the central goal, especially around 180°

The phase-difference between the perturbation field and uniform field is, respectively, calculated when the conductivity of the center perturbation increases from 0.2 to 2.0 S/m. The result is shown in Fig. 5. It shows that the phase-difference have a noticeable increase when the disturbance has higher conductivity.

**Fig. 6:**
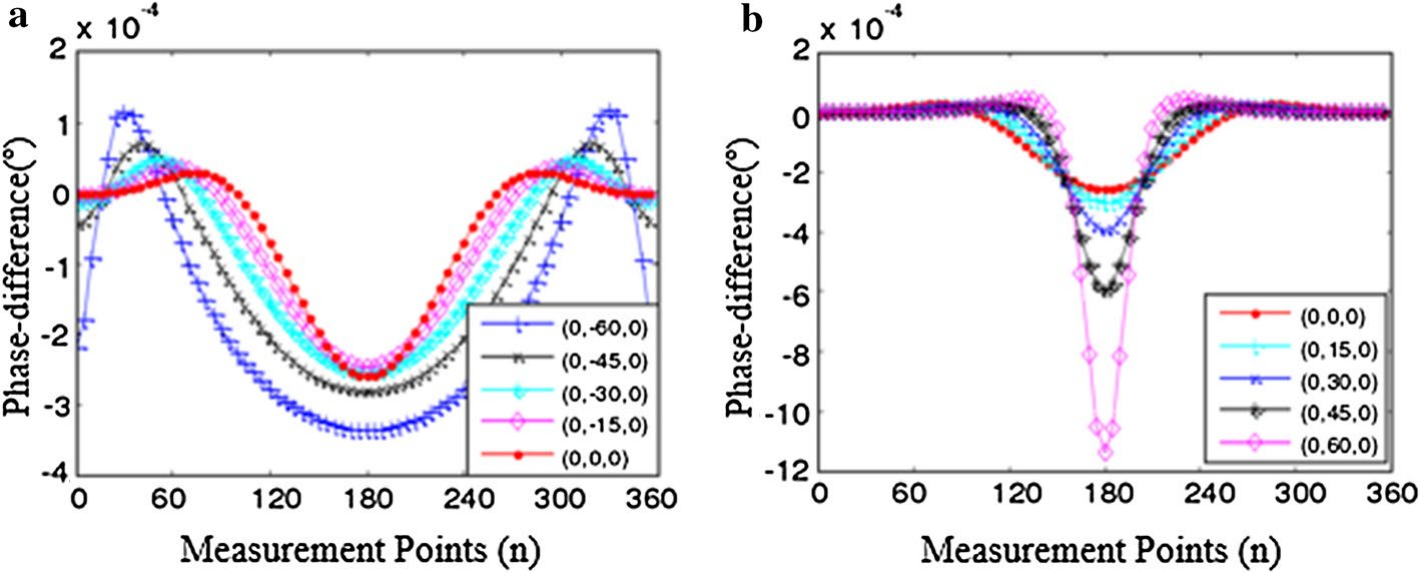
The phase-difference distribution when the goal moves along *Y*-axis. The motion paths of goal on the *Y*-axis is divide into two parts: the same side of the drive coil (the negative axis) and the opposite of drive coil (the positive axis). **a** The goal on the same side of the drive coil. **b** The goal at the opposite of the drive coil

**Fig. 7:**
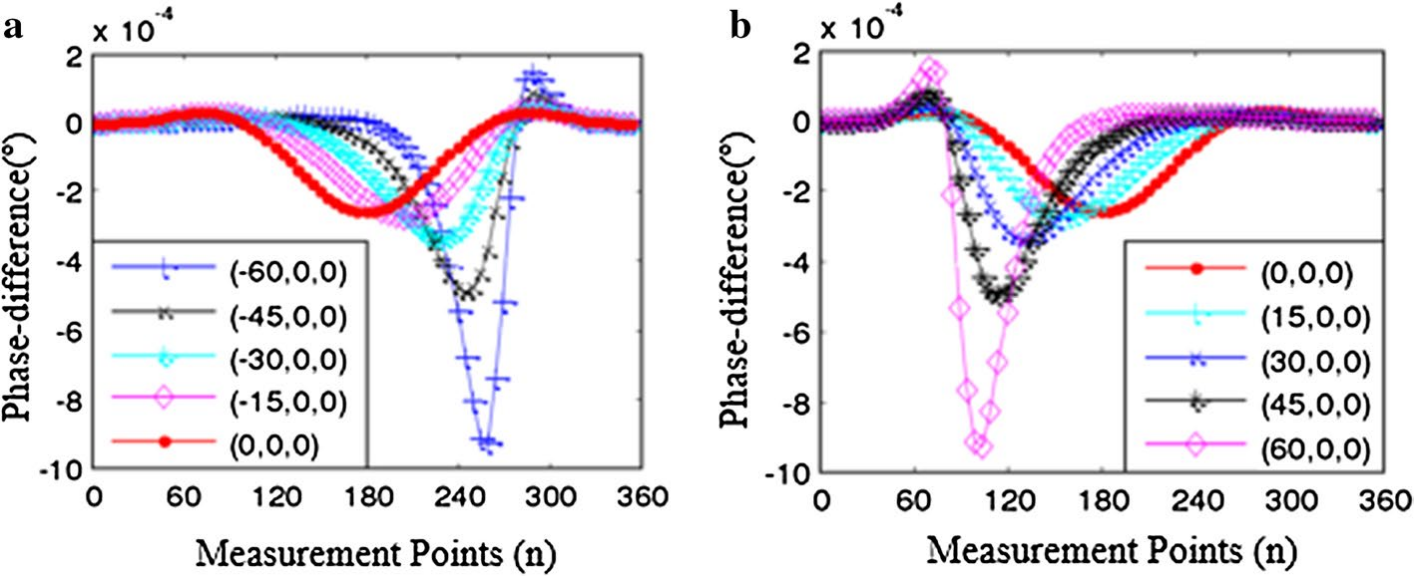
The phase-difference distribution when the object moves along the *X*-axis. **a** The goal moves on the negative *x*-axis. **b** The goal moves on the positive *x*-axis

The axis sensitivity simulation model is designed to analyze the sensitivity of SMIT when the goal at different positions. The coordinate points of goal on the axis are typical points which have the same spacing. In this model, boundary phase-difference are calculated when perturbation moves along the transverse direction and longitudinal direction. The result of the *Y*-axis (longitudinal) sensitivity analysis is shown in Fig. 6. The perturbation (1 S/m) moves from (0, − 60, 0) to (0, 60, 0) in the range from (0, − 100, 0) to (0, 100, 0), and the size of step is 15 mm. A set of data about phase-difference can be obtained when perturbation moves one step. The value of the phase-difference has a decline when the perturbation moves away from the drive coil along negative *Y*-axis (see Fig. 6a). On the other hand, the goal has an evident impact on the checkpoints, especially when the goal close to the boundary (see Fig. 6b). The phase-difference has a big change at the scope of 90°–270° according to the analyses above. The results of *X*-axis sensitivity analysis are shown in Fig. 7. The perturbation moves from (− 60, 0, 0) to (60, 0, 0), and the step size is 15 mm. The phase-difference has obvious change when the goal gradually close to the checkpoints. The phase-difference also has an obvious amplitude in the range of 90°–270°.

The effect of the perturbation on the sensors is uneven based on the simulation result. In the SMIT system, the limited amounts of sensors need to be placed on the high detection sensitivity region. The threshold is defined to differentiate the effectiveness of all checkpoints. In this paper, the value of the threshold is defined as 1/6 of the phase-difference peak. A checkpoint can be defined as effective when the absolute value of phase-difference is large than the threshold; otherwise, this checkpoint will be ignored. Moreover, the sensitivity of checkpoints depends on energy ratio (the ratio between the energy of measuring point and the highest energy). The energy ratio of measuring points will be a standard to decide whether the loss of measuring points will influence the quality of the image. The energy ratio of all checkpoints are collected when the perturbation locate on the representative detecting points [(− 75, 0, 0), (75, 0, 0), (0, − 75, 0), (0, 75, 0), (0, 0, 0)] of the imaging area. The low sensitivity points will be defined as almost no impact on the quality of images when the energy ratio is less than 10%. The ratio value of all checkpoints has been calculated, and the results are shown in Table 7. The high sensitivity sensor positions almost locate on the semicircle opposite the exciting coil. The total energy ratio of sensors locating on this semicircle is greater than 90%. Therefore, the semicircle sensor array is designed to meet the needs of measurement.

**Table 7:** The energy proportion of the effective detecting points of the total energy

Perturbation location	Distribution of efficient point	Efficient value energy ratio (%)
(0, 0, 0)	115°–245°	98.56
(− 75, 0, 0)	245°–275°	97.88
(75, 0, 0)	85°–115°	97.65
(0, − 75, 0)	0°, 95°–265°, 360°	89.7
(0, 75, 0)	170°–185°	98.11

The effective measuring region almost locate on the opposite side of drive coil. The semicircle detection area can receive 90 percent of total energy when the goal at five different places

### SMIT hardware system

**Fig. 8:**
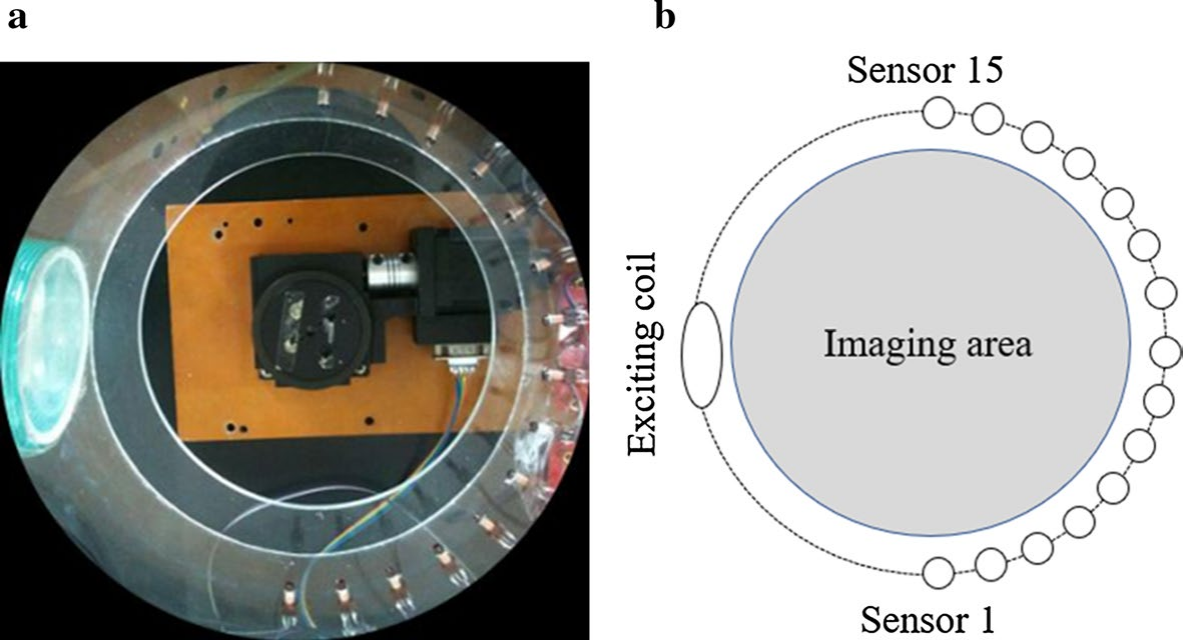
The drive coil and the sensor array of the SMIT system. The drive coil and sensor coils are fixed on the circular acrylic plate. **a** The experimental facilities of the sector sensor array and the exciting coil. **b** The sketch of the sector sensor array and the exciting coil

The SMIT system is designed in this paper. The system includes an exciting coil and a semicircle sensor coil array (Fig. 8a), and the imaging region locates between them. A controllable rotating platform is placed on the bottom of the imaging region, and a motion card can control the rotating platform. The inductance of the drive coil is 12.17 μH, and the capacitance is 32.5 pF. The sinusoidal signal (8 MHz) is jointed into the drive coil to generate resonant in the exciting coil. The drive coil working in resonant frequency can increase the signal-to-noise ratio of the main field [17]. The sensor coils are placed the periphery of the half-cylindrical opposite the drive coil. All sensor coils work under resonant frequent, and the resonant can improve the signal-to-noise of measured voltage. The sensor array contains fifteen commercial sensor coils, and the inductance of every sensor coil is 11.88 μH, and the capacitance is 33.3 pF. In the process of measurement, the DG1022 function generator generates a sinusoidal drive signal (20 V and 8 MHz).

The phase-difference detecting is completed using AD8302 (Analog Devices, Inc.) which is a fully integrated system for measuring the phase-efference and amplitude ratio between two input signals. In the signal detection, the function of phase discriminator in AD8302 is used. The output results of chips are essential information for image reconstruction. The NI PCI6221 (National Instruments, Inc.) data acquisition card is used to realize the functions of multiplexer control, signal generation and data acquisition. The built-in LabView functions are used to measure the output amplitude change of AD8302. The region of Imaging of the SMIT system is a circular space with a radius of 100 mm. The exciting coil and sensor array are placed on the edge of the circular imaging region. The sketch of ROI is shown in Fig. 8b.

**Fig. 9:**
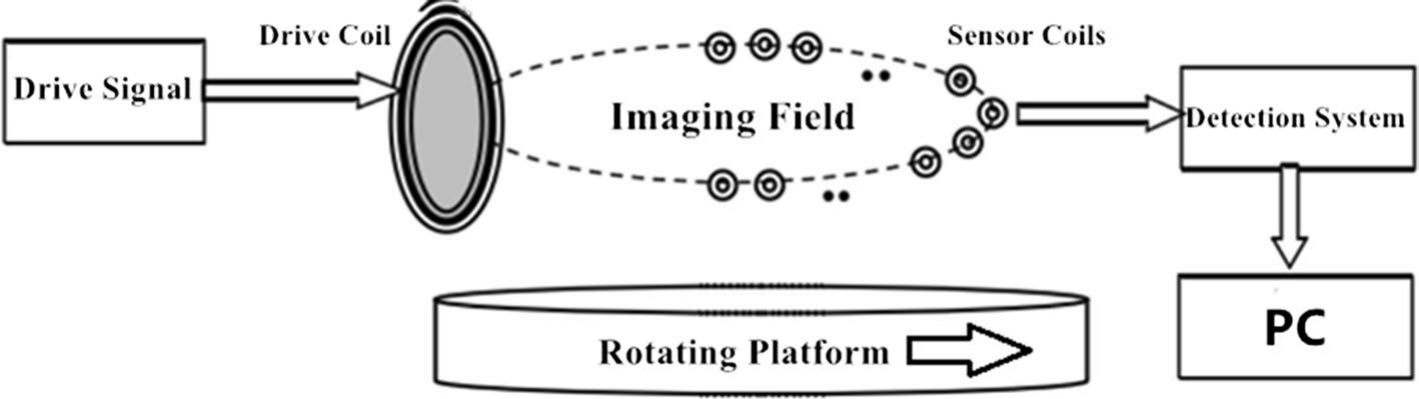
The system block diagram of SMIT. During the measurement, the rotating platform rotates the goal to complete scanning measurement. The process of rotating and detection is controlled by the computer

The simplified system block diagram of the SMIT system is shown in Fig. 9. The operating principle of the SMIT is as follows. The drive current signal is injected into the exciting coil to generate the main field. The 15 sensor coils in the sensor array convert the electromagnetic signal into a voltage signal. The induced voltage signal is transmitted to signal processing channels. The information of phase-difference from the output of chip will be collected and stored. The rotating platform is used to obtain the detection results of the complete measurement. The object is placed on the rotating platform, and the platform does a *θ*-degree (a step) rotation when the system finishes single step measurement. The number of rotation steps can be set to an integer (*N*) by the requirement of the experiment. Therefore, the number of detection values of a set of imaging data is 15 × *N* (*θ* × *N* = 360°).

The semicircular sensor array and the independent drive coil make the SMIT system different from previous MIT systems. The feasibility of structure design receives the theoretical demonstration based on the simulation result. The actual test is performed to verify the sensitivity and stability of the system at the same time. The measuring data is used to calculate the sensitivity of the system when the imaging field contains a sufficiently large goal. The test result contains all the sensor combinations (15 × *N*). In the process of measurement, the sinusoidal voltage signal is injected into the exciting coil to provide a stable main field, and the component of the injected signal is connected to the phase-difference detection system as the synchronous reference waveform.

**Fig. 10:**
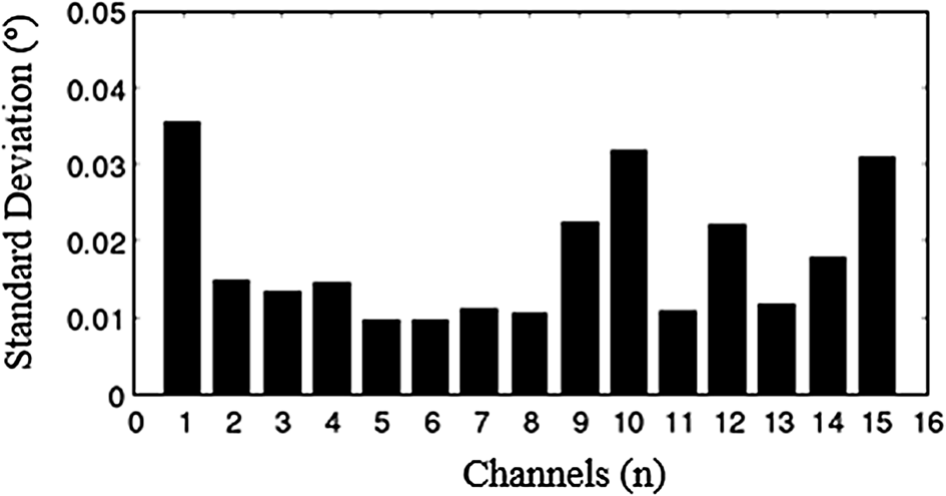
The standard deviation of every sensor coil. The error has been measured when the imaging region has no goal. The results show that the system is stable

**Fig. 11:**
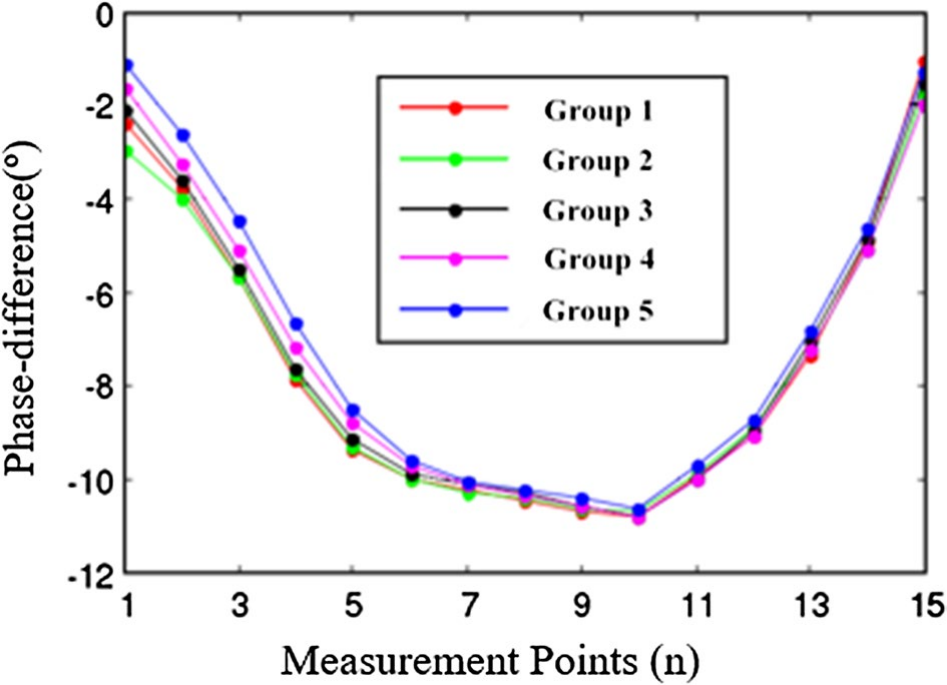
The measured phase-difference of all channels in goal experiment. In the goal experiment, the goal is placed in the center of the imaging region. The experiment is repeated five times, and the variation trend of phase-difference are identical

In the sensitivity test of the system, the perturbation is a 160 mm diameter cylinder which includes the saline solution with the height of 100 mm, and the conductivity of the saline solution is 3 S/m. Thirty repeated measurements are done in each coil. To further verify the stability of our system, the uniform field experimental data also have been measured, respectively. The measurement process of 40 sets of sensor data cost 40 min. The standard deviation of every sensor coil is shown in Fig. 10. It shows that the minimum appears in coils 5 and 6 (0.009), the maximum (0.035) appears in coil 1. The standard deviation of twelve coils in the sensor coil array is less than 0.03; the total standard deviation of all sensor coils is 0.018. The stability of the system can meet the requirement of the measurement. In the perturbation test, the measurement result is revised by the uniform field data (the imaging data is only the impact of goal on the phase-difference). Five sets of revised results of perturbation test are shown in Fig. 11. It shows that the larger phase-difference locate from coil 6 to coil 10. The phase-difference values are very satisfying by the calculative result above. The distribution rule of goal test data is basically in line with perturbation field simulation data (see Fig. 5).

This section describes the operating principle, sensor, hardware, software, and image reconstruction of the system. The feasibility of using sector sensor coils array is clearly shown in this test. The system has good SNR indicates and the stability according to the contrast of the uniform field test data and the perturbation field test data. The image reconstruction work is done with MATLAB software.

## Data Availability

The research data related to the design and simulation results are included within the article. For more information on the data, contact the corresponding author.

## Abbreviations

MIT: magnetic induction tomography
ROI: region of interest
SMIT: sector sensor array magnetic induction tomography
CG: center goal
MG: marginal goal
PSNR: peak signal-to-noise ratio PSNR
FEM: finite element method
